# The Impact of Low-Carbon City (LCC) on Elderly People’s Health: Evidence from a Natural Experiment in China

**DOI:** 10.3390/ijerph19159424

**Published:** 2022-08-01

**Authors:** Shaohong Mu, Weixiu Li, Muhammad Mohiuddin

**Affiliations:** 1 School of Accountancy, Shandong University of Finance and Economics, Jinan 250014, China; shaohongmu@163.com; 2 Longshan Honors School, Shandong University of Finance and Economics, Jinan 250014, China; liweixiu@mail.sdufe.edu.cn; 3 School of Finance, Shandong University of Finance and Economics, Jinan 250014, China; 4 Faculty of Business Administration, Laval University, Quebec, QC G1V 0A6, Canada

**Keywords:** new urbanization, low-carbon city, construction, haze pollution, elderly health

## Abstract

Rapid urbanization has increased haze pollution, affecting the health of elderly people. This study uses low-carbon city (LCC) data and examines the effects of LCCs on improving the health of elderly residents. Our main purpose is to explore the following question: Can the new urbanization model presented by the LCC alleviate haze pollution and enhance the health of middle-aged and elderly people? This study uses data from the China Health and Retirement Longitudinal Study (CHARLS) and the 2012 LCC pilot to explore whether the LCC can alleviate haze pollution and improve elderly people’s health. The study found that the building of LCCs can reduce blood pressure, improve vital capacity, decrease obesity, and improve memory levels, including short-term and long-term memory. The building of LCCs also reduces the probability of being exposed to haze pollution by increasing the city’s green total factor productivity and the use of green technologies. The study concludes that elderly people received health dividends as a result of the enhancement of living conditions, transportation, and medical support in the LCCs.

## 1. Introduction

China has observed tremendous levels of urbanization, from less than 20% in the 1980s to nearly 60% today [1], and the urbanization rate is expected to reach 75% by 2030 according to the China Urbanization 2.0 report [2]. This has been accompanied by significant improvements in medical and health conditions, an increase in average life expectancy, and a decrease in infant mortality. Nevertheless, rapid economic growth, excessive urbanization, and extensive economic development have created serious pressures on urban infrastructures, living conditions, and environmental pollution [3]. They also pose a threat to the health of residents [4].

As a typical case of environmental pollution, “haze” occurs when the PM_2.5_ emitted by the economic and social activities of a high-density population exceeds the capacity of atmospheric circulation [5]. More than 50% of China’s population has been exposed to PM_2.5_ in recent years. In 2018, the average concentration of PM_2.5_ in China was 39 g/m^3^, 9.3 percent lower than in 2017. The number of hazy days in 2018 was 20.5, which is 7.1 fewer than in 2017, according to the Meteorological Bulletin of the Atmospheric Environment [6]. There is no denying that the effect of the pollution control of the Chinese government is remarkable; however, the haze exposure rate is still significantly higher than those of OECD countries, which are lower than 20% [7].

Haze seriously affects the health of residents. Exposure to PM_2.5_, both long-term and short-term, increases residents’ chances of developing chronic diseases [2]. Because PM_2.5_ has small particle size, large surface area, and the ability to carry dangerous compounds in the atmosphere for an extended period of time, it has a negative influence on public health and the atmosphere [8]. Authors [9] have found that the death toll due to ozone was 4700, while the number of deaths caused by PM_2.5_ was as high as 130,000. Moreover, people aged 65–99 had lost 1.1 million years of life as a result of exposure to PM_2.5_. These findings highlight that haze is more lethal than other forms of air pollution, and that the elderly are more sensitive to haze than other age cohorts. According to statistics, the elderly population in China accounted for 18.1% of the total population in 2019, and this proportion is expected to rise to 20% by 2030. By then, China will enter a heavily aging society. In addition, there will be accelerated growth of the proportion of the population that is aging [10]. Therefore, it is important to assess whether the building of “low-carbon cities” (LCCs) has been effective in mitigating the effects of haze pollution and improving the health of the elderly.

There is great heterogeneity in pollution control in different cities. Generally speaking, the level of urban economic development, innovation ability, and technological level will affect urban pollution control. The use of green total factor productivity and green technology in cities will greatly reduce the possibility of smog pollution.

The Reform Commission (NDRC) launched three stages of LCCP projects in 2010, 2012, and 2017. In 2012, in order to further carry out the target of “building a beautiful China”, the NDRC launched the second project in 29 provinces and cities, expanding the scope of LCCP projects [11]. A low-carbon city promotes complete low-carbon urban development by improving energy efficiency, adjusting energy structure, transitioning high-carbon industries to low-carbon industries, and allocating more environmentally friendly resources. Low-carbon cities’ new style of urbanization can relieve pressure on the urban environment, reduce hazy weather, and promote green development. The research on haze and the different types of health problems of the elderly is extensive. Smog particles suspended in the air can damage heart and lung function. In an analysis of PM_2.5_ and elderly cardiovascular disease, the authors of [12] observed that chronic exposure to PM_2.5_ is associated with a variety of cardiovascular diseases that are a direct cause of death and reduce elderly people’s mobility. In a blood pressure test survey of 12,665 elderly residents aged 50 and over, the probability of PM_2.5_ raising 10 μg/m^3^ blood pressure increased by 1.14%, with obesity strengthening this trend [13]. It has been demonstrated that haze and obesity are also related [9]. In terms of lung function, the authors of [14] evaluated the effect of PM_2.5_ concentration on the number of patients in the local respiratory clinic and found that when smog particles reached 200–400 µg/m^3^, the number of patients surged. It is even reported that 48.6% of the Chinese population (nearly 100 million people) suffer from obstructive pulmonary disease (COPD), and 18.7% of COPD deaths are attributable to environmental PM_2.5_ [2]. The authors of [15,16] even posited that PM_2.5_ is an influencing factor of lung cancer. In addition to the abovementioned cardiopulmonary effects on the elderly, some scholars have concluded that smog can also damage their memory and cognitive ability [17].

The urban living and health issues have become a focus of academics and policymakers alike. Traffic congestion and the urban heat island effect pose challenges to the human settlement environment. The increase in urban construction land has occupied green areas within cities. While this reduction of green space has a negative effect on all people’s physical and mental health [18,19], the elderly and children are most vulnerable to these effects. Furthermore, long-term sedentary behavior and continuous stress are closely related to the modern urban lifestyle and increase the probability of the elderly suffering from mobility-reducing disorders such as diabetes, obesity, and depression [20].

Addressing the ecological consequences and health hazards caused by rapid urbanization has become a widely discussed issue. Aiming to reduce regulatory costs and increase regulatory efficiency, developed countries have been exploring environmental regulations since the 1980s. After previously employing command-controlled environmental governance tools, China introduced an emissions trading system in the 1980s and began to try command-based and market-based tools. Policy evaluations of the low-carbon economy have gradually become a research highlight. Some scholars have assessed the impact of low-carbon economic circle policies on air pollution [21,22,23]. The authors of [3,24] also investigated the impact of low-carbon zone policies on infant mortality. These studies have concluded that low-carbon zone policy is effective, and it both improves air quality and reduces infant mortality.

However, some authors [25] found that environmental regulations did not reduce the infant mortality rate significantly, did not change levels of water pollution, and only slightly improved air pollution. They asserted that the legal system in developing countries is relatively weak and held that to achieve the desired environmental regulatory effect, it is necessary to increase public support. In 2010, China launched LCCs as pilot projects. Can the new urbanization model presented by the low-carbon route alleviate haze pollution and enhance the health of middle-aged and elderly people? This study uses data from the China Health and Retirement Longitudinal Study (CHARLS) and the 2012 LCC pilot to answer the above research question, which has not yet been fully explained by the extant research.

This study investigates the mechanism of new urbanization and aims to improve the health of elderly people. Other possible channels for improving residents’ health, such as the Chinese government’s green infrastructure and medical investments, were examined, including the urban green space area, use of renewable energy, number of hospitals (clinics) and vacant hospital beds, and health and family planning investment [26,27].

The study makes the following research contributions. First, using the difference-in-difference (DID) method, we treat China’s construction of LCCs as a quasi-natural experiment. Meanwhile, through a rich physical examination and by adopting mental health indicators, the study evaluates how these LCCs have improved the health of elderly people. The study effectively solves endogenous problems while also systematically investigating the physical and mental health problems of elderly people.

Second, our mechanism analysis investigated the effect of the construction of LCCs on improving haze, and the mechanism behind their significant effect on haze improvement was explained from the perspective of government environmental regulations and enterprise innovation [28,29]. Other possible channels through which LCC construction improves residents’ health were also examined. By investigating the cities’ health promotion effect through multiple dimensions, we provide a broader research perspective for the subsequent evaluation of low-carbon economic policies.

Third, in contrast to the research of [3] on the effects of environmental regulations in India, the present study posits that with the support of adjustments in personal exercise habits, enterprise innovation, emission reduction and pollution control, government environmental regulations, and financial investment, developing countries can also achieve the expected effects of environmental regulation even if their legal systems are not yet mature. These efforts can also enhance the physical and mental health of the elderly, who are at high risk of disease.

## 2. Policy Background and Research Hypotheses

Global climate change has become severe in recent years, and the ecological environment has faced periodic catastrophes [1]. Carbon emissions have emerged as a major source of global warming, and the development of low-carbon cities has progressively emerged as an important step in mitigating global warming and improving environmental quality [30].

The LCC has become the new standard for China’s low-carbon economic development and new urbanization. The pilot LCC projects in China were divided into three batches [31]. The first batch was in 2010, and a total of five provinces and eight cities were included in the pilot scope. The emission reduction targets were that energy consumption per unit of GDP dropped by 16%, unit carbon dioxide emissions fell by 17%, and non-petrochemical energy accounted for 11.4% of the primary energy consumption targets. Tourism cities and industrial cities differ greatly in their emission reduction elasticity; therefore, this study does not consider the first batch of LCCs. The third batch was the district and county pilot in 2012. On the one hand, the effects of this policy pilot are too new to identify based on current data. On the other hand, the district and county data are difficult to obtain; therefore, the third batch of LCCs was also not considered in this study.

The second batch was introduced in 2012, involving 26 prefecture-level cities and one province. The targets included 14 indicators, such as total carbon emissions and carbon emissions per unit of GDP. Further, all pilot cities were required to formulate corresponding measures according to their own planning. The emission reduction plan clarified the emission reduction tasks and time node, aiming to evaluate whether they could be used for the promotion of similar cities. In addition to each city’s own task setting, the national document outlined low-carbon development plans, which included establishing a low-carbon industrial system characterized by low-carbon, green, environmental protection, and recycling; building a greenhouse gas emission data statistics and management system; and advocating low-carbon green lifestyles and consumption patterns. The document indicated that under the central planning outline, pilot cities combining their own city’s positioning with the national plan would actively play a role in the development of the low-carbon economy and take a new path toward urbanization.

**Figure 1:**
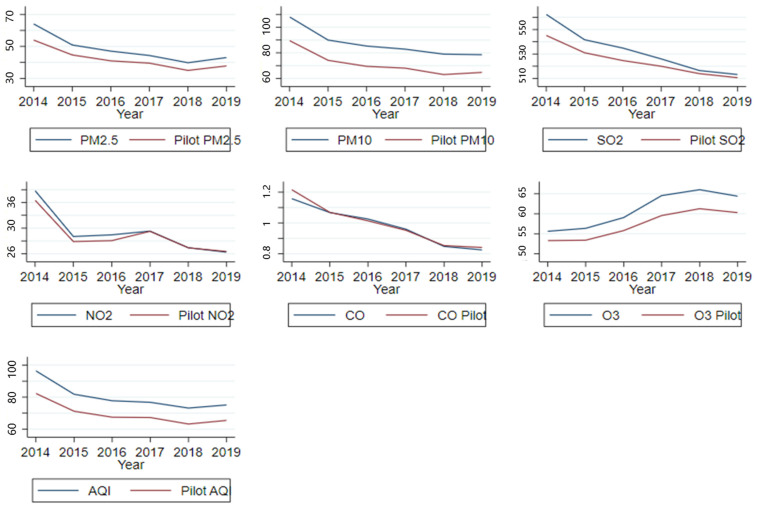
Air pollution trends in pilot and non-pilot cities.

Figure 1 depicts the annual average air pollution indicators of low-carbon pilot cities and non-pilot cities from 2014 to 2019. The LCCs had both lower PM_2.5_ levels than the non-pilot cities and lower levels of other types of pollutants such as PM_10_, SO_2,_ NO_2_, CO, and O_3_. The air pollution index (AQI) also showed the same pattern. Although there was a large difference in the air pollution between pilot and non-pilot cities, due to our use of the DID method for the assessment of LCCs’ potential to reduce PM_2.5_ and other pollutants, it was only necessary to meet the pollution trend of the treatment and control groups before we could consider the introduction of the policy as being consistent. Though the basic conclusions of a parallel trend can be drawn from Figure 1, we used the strict regression drawing method to verify the parallel assumption; otherwise, the policy evaluation would have been biased. Similarly, it was necessary to meet the basic assumption of the DID method in the assessment of changes in the health status of elderly people in the benchmark regression.

If we can verify that building LCCs can reduce haze pollution and thereby enhance the health of the elderly, we must also consider how LCCs reduce haze pollution. We took the Changzhou government documents related to the third batch of LCCs as an example. The specific aims of the Changzhou government were, first, to establish and improve the city’s unit GDP carbon dioxide emission reduction target and total carbon emission control target responsibility assessment and evaluation mechanism; and second, to formulate measures to identify low-carbon enterprises in Changzhou and encourage and guide low-carbon development among enterprises, cultivating a group of leading low-carbon businesses with distinctive advantages. The city aimed to identify 160 low-carbon emission enterprises at different level of development. What can we discover from the details of these documents?

First, the local government introduced a promotion and evaluation incentive system for emission and pollution reduction, which embodied not only the official logic of the promotion and incentive system for Chinese officials but also the firm determination of the local government to reduce environmental pollution. We have reason to believe that the personnel of various administrative departments took certain measures to obtain further upward mobility opportunities; for example, the environmental and ecological protection departments adopted more stringent environmental regulations, which was expected to increase pollution control and reduce undesirable pollutants. Thus, we hypothesized that the pilot cities reduced haze pollution through the improvement of government-level environmental regulations, diminishing the probability of the elderly being exposed to haze and improving the health of this group:

**Hypothesis** **1.** *After the introduction of the low-carbon construction pilots, the government reduced haze by increasing the intensity of environmental regulations and improving the health indicators of the elderly population.*

Second, in pilot cities, leading enterprises with low-carbon development potential are nurtured through technical support and tax incentives from the government, at the same time as being subjected to stricter environmental regulations. This is in line with the increasingly strict environmental regulations mentioned in the environmental porter hypothesis. Enterprises reduce production and operating costs through the use of existing technologies to improve efficiency and develop new technological innovations. The government achieved a win-win situation in terms of its environmental regulations and economic growth, which was reflected in the green total factor productivity (GTFP), green technological progress (GTC), and green technology utilization efficiency (GTE) at the city level. This suggests that LCCs can achieve sustainable development by motivating companies to enhance existing technology efficiency and technological innovation. Therefore, we hypothesized that LCCs can reduce haze pollution by increasing GTFP at the city level, thereby improving the health of elderly people:

**Hypothesis** **2.** *Enterprises in low-carbon cities improve the city’s green total factor productivity, green technology progress efficiency, and green technology utilization efficiency through technological innovation; reducing the probability of elderly groups being exposed to haze and improving their health status.*

Finally, in addition to increasing the intensity of environmental regulations and giving enterprises more technical and tax incentive support, green public investment and green infrastructure development are other powerful measures to reduce emissions and pollution. The supply of green public goods at the government level is another effective way to reduce haze, including the construction of urban green space and the use of green renewable energy (gas and electric buses) [32,33,34]. Expanding green space can both absorb suspended pollutants, such as PM_2.5_, in the air and increase the opportunities for elderly people to go out and exercise. Similarly, the improvement of a green public transportation system can both reduce the number of private car trips and vehicle emissions and increase the chance of green travel for elderly groups, which provides a further theoretical basis for the predicted improvement of the health status of the elderly. Moreover, as the environment and health are closely related, in addition to increasing its investment in green infrastructure, the government increased the number of hospitals and vacant hospital beds to enhance residents’ living environment. At the same time, the government increased its expenditure on health and family planning, which could enhance the health status of elderly people in the pilot cities. Given this, we hypothesized:

**Hypothesis** **3.** *The pilot city government’s increase in green public investment, green infrastructure construction, and investment in health and family will improve the health of elderly people.*

## 3. Data and Identification Strategies

### 3.1. Data Overview

This study’s empirical analysis used three phases of data from 2011, 2013, and 2015 from the CHARLS database. This database is a set of high-quality micro-databases collected by Peking University that include the basic personal information, economic status, health status, and medical service use of families and individuals of middle-age and over 45 years old. The advantages of using the three-phase CHARLS data were that the database includes a variety of health indicators, including physical examination indicators, subjective questions, and quizzes, which can facilitate the examination of the health status of middle-aged and elderly groups from multiple dimensions. Moreover, the pilot policy examined by this study was initiated in 2012, and the three-phase balanced panel data can eliminate the influence of factors that change with individuals but not with time, therefore providing the DID setting for this research. In addition, we collected data on the low-carbon policies of pilot cities, which were sourced from the official websites of local governments.

Further, this study’s mechanism analysis used 2008 to 2016 data from 281 cities at the prefecture level and above. The city-level explanatory variables included the square area of green space, number of green buses, health and family planning investment, number of hospitals and vacant hospital beds, and various pollutant emissions and decontamination rate data derived from the CEIC database. Control variables at the city level included population density, financial surplus (fiscal revenue—fiscal expenditure), unemployment rate, capital per capita, proportion of secondary industry to GDP, proportion of tertiary industry to GDP, proportion of industry value-added to GDP, proportion of foreign investment to GDP, and GDP growth rate, which were also derived from the CEIC database; weather variables derived from the China National Meteorological Administration; the air flow coefficient derived from the European Weather Data Center; PM_2.5_ data collected from Columbia University; green invention patents and green utility patents derived from the Chinese Intellectual Property Office Patent database; and the urban innovation index from Fudan University.

The level of environmental regulation was calculated using the entropy method. Determining the weight of each indicator through this method can not only help avoid the randomness and presumption of unavoidable subjective weighting but also effectively solve the problem of information overlap between multiple indicators. The entropy method was used to calculate the strength of the government’s environmental regulations after determining the expected outputs (five indicators including SO_2_, smog, solid waste, sewage, and garbage removal rate) and undesired outputs (four indicators including SO_2_, sewage, smog, and PM_2.5_ emissions).

Referring to [35], we constructed a set of production possibilities, including the expected and undesired outputs, and calculated the Malmquist–Luenberger index using the non-radial SBM directional distance method. The input indicators included labor input, capital stock, and energy consumption; the desirable outputs included GDP; and the undesirable outputs included SO_2_, sewage, and smog emissions. GTC and GTF are decomposed according to the dynamic changes of GTFP, which measured technological innovation and the use efficiency of existing technology, respectively, to better observe the mechanism of the green development effects of LCCs.

### 3.2. Identification Strategy

#### 3.2.1. Benchmark Regression

We used the DID method to identify the physical and mental health effects of the LCCs, as follows:

(1) $$Y_{it}=\alpha +\beta Pilot_{it}\times Treat_{it}+\delta X_{it}+\tau_{t}+\omega_{i}+\varepsilon_{it}$$

In this equation, $Y_{it}$ represents a group of individual-level outcome variables, including indicators of blood pressure, vital capacity, and weight to evaluate physical health, as well as self-rated memory and personal memory test scores, which measured short-term instantaneous memory and long-term persistent memory to evaluate mental health. These two-dimensional health indicators offered a comprehensive overview of the health status of middle-aged and elderly people. $Pilot_{it}\times Treat_{it}$ is the interactive item of the LCC pilots of the treatment group and the dummy variables before and after the initiation of the pilot. The coefficient of the interactive item β is the core coefficient of concern in this study and represented the difference between the result variables of the elderly groups in the low-carbon and non-pilot cities. Whether LCCs can affect the physical and mental health of elderly people in the short- and long-term will be discussed later.

The year 2011 is considered as before the treatment ($Treat_{it}$ = 0), and 2013 and 2015 were regarded as after the treatment ($Treat_{it}$ = 1), since the second batch of LCC construction was in 2012. $X_{it}$ represents a set of individual-level covariates, including demographic variables such as age, gender, and marital status; socioeconomic variables such as average household income; and health variables such as self-rated health, exercise intensity, whether individuals had smoked or drunk alcohol recently, whether they had been to hospital in the last month, whether they had been hospitalized in the last year, and whether they had medical insurance. $\tau_{t}$ is the fixed effect of the year, while $\omega_{i}$ is the individual fixed effect. Using the two-way fixed effect of the individual and year can better control the unobservable factors at the individual level that do not change with time; thus, the average treatment effect in this study was based on the balance of the panel data. $\varepsilon_{it}$ indicates a disturbance. It should be noted that the model did not control for the fixed effects of communities or cities since factors that do not change with time were absorbed by the individual fixed effects. Meanwhile, variables that do not change with time, such as gender and education level, were not included in the control variables.

#### 3.2.2. Semi-Parametric Difference-in-Difference (SDID)

This processing method makes DID results more credible. The credibility of DID results depends on the assumption of the common trend, which means that the health status trend of the elderly population in pilot cities was the same as in the non-pilot cities. However, it was impossible to directly test the common trend premise owing to the few years of micro-data available. Though this study controlled for individual fixed effects, there was still a possibility of the failure of the common trend due to time-varying factors. In particular, there were only 11 cities in the treatment group and 83 cities in the control group, which could affect the validity of the results due to accidental factors or the impact of other policies. Therefore, we conducted a series of tests as follows.

We used the re-weighted SDID method proposed by the author of [36] (ABSDID) to test for robustness. In the case of two-phase balanced panel data, this method is more balanced by the characteristics of the weighted treatment group and the control group. Finally, the policy effect was examined by comparing the changes in the outcome variables of the weighted treatment and control groups over the two periods in order to ensure the conclusions were credible even if the common trend was not fully satisfied. The average treatment effect of SDID was calculated as follows:

(2) $$E\left[\frac{\Delta Y_{t}}{P(d=1)}\times \frac{d_{t}-\pi (X_{b})}{1-\pi (X_{b})}\right]$$

where $d_{t}$ represents whether it was a treatment group at time *t*, *P*($d_{t}$ = 1) represents the probability of the treatment group, and $\pi (X_{b})$ represents weights for Abadie, which can be calculated by linear probability model $\pi (X_{b})$ = *P*($d_{t}$ = 1|$X_{b}$). According to [37], SDID is a credible research method since common trends cannot be guaranteed. Therefore, this study used the ABS-DID method for further verification.

#### 3.2.3. Propensity Score Matching (PSM)-DID

As mentioned above, there was a large gap between the number of cities in the treatment and control groups, meaning that systematic differences in the economic and social backgrounds of the treatment and control groups could be found. Consequently, the propensity-scores-matching difference-in-difference method (PSM-DID) was further used to test the robustness of the benchmark results.

Whether a city can successfully apply to be a LCC pilot depends on its socioeconomic background indicators. We adopted the PSM-DID method [38] to evaluate the policy effect of LCC construction. To begin with, we collected the 2010 urban-level social and economic development indicators, applying the PSM method to match the pilot cities with non-pilot cities. After matching, we deleted the cities that did not meet the common trend hypothesis and analyzed the average treatment effect (ATE) for the elderly groups. The matching covariates included the proportion of secondary industry to GDP, proportion of tertiary industry to GDP, unemployment rate, capital per capita, population density, urban innovation index, financial surplus, and GDP growth rate, which were set as a possibility index that affected whether the city could be selected as an LCC.

#### 3.2.4. Falsification Test

Given that the quantity of the control group was small, to reduce the bias caused by the imbalance between the treatment group and the control group, the cities in the treatment group and the control group were randomly disrupted to test whether there were other impact effects that could pollute the results. Eleven LCC pilots were randomly introduced in the year 2012, and the regression model was based on Equation (1). We repeated the regression 500 times, depicting the regression coefficient β density graph. If the results were robust, the density distribution of the simulated coefficient β would be concentrated around 0, and the true coefficients would be significantly different from the β density distribution. As per the authors of [39,40], we also used a falsification test to further verify the credibility of our results.

In addition, we specifically screened the more appropriate control group at the policy level in order to eliminate the effects of other related policies and ensure the rigor of the conclusions. The identification strategy of the mechanism analysis is explained in more detail below.

### 3.3. Data Description

In this study, the benchmark regression used the balanced panel data of the CHRALS from 2011, 2013, and 2015, with a total of 30,024 effective samples. The mechanism analysis used the balanced panel data at the prefecture level and above from 2008 to 2016. There were 2529 actual effective samples after matching according to the PSM method. The year 2011 was selected for empirical analysis (Panels A and B), and 2010 was chosen for the mechanism analysis (Panel C) to unify the descriptive statistics of the average difference between groups since the empirical testing used data from the second batch of LCCs in 2012, and the mechanism analysis used data from two batches of LCCs in 2010 and 2012. The descriptive information of the specific variables is shown in Table 1.

Panel A and Panel B are descriptive statistical analyses of the CHARLS data. The health status of the elderly population in the pilot cities was statistically significantly better than that of those living in non-pilot cities, thus there was a threat that the DID common trend may not be satisfied. Therefore, we also used ABS-DID and PSM-DID methods to mitigate this threat. Panel C is a mix of city-level variables, which includes PM_2.5_, dependent variables, and other macro-control variables. There was no significant difference between the pilot cities and non-pilot cities in terms of PM_2.5_, environmental regulation, and GTFP.

In summary, we aimed to demonstrate that the building of LCCs can improve the health of elderly groups by reducing haze pollution by successfully identifying this effect and undertaking a series of robustness tests and endogenous discussions to verify Hypotheses 1 to 3.

## 4. Empirical Testing

### 4.1. Impact of Low-Carbon Cities on the Health of Elderly

According to model (1), which used three phases of data from 2011, 2013, and 2015, the estimation results of LCCs on the physical and mental health effects are shown in Table 2.

Columns (1)–(3) show that the construction of LCCs reduced the blood pressure and weight of the elderly residents in pilot cities by 1.8% and 1.7%, respectively, when compared with elderly residents in cities that had not implemented the policy. Moreover, the observed increase in their lung capacity was more than 14%. Meanwhile, the results in Columns (4)–(6) reveal that LCCs increased elderly residents’ self-rated memory by 9% compared with non-pilot cities and increased their short-term memory and long-term memory by 11.6% and 0.8%, respectively.

The basic regression results indicated that the construction of LCCs can effectively improve the physical and mental health of elderly residents. The possible reason is that LCCs reduce the probability of elderly people being exposed to haze pollution. At the same time, government fiscal expenditures in LCCs are inclined toward green public infrastructure and public health investment, thereby also improving the health of elderly residents. The above possible explanations were verified in our further mechanism analysis.

### 4.2. Robustness Testing

#### 4.2.1. Abadie SDID Weighted Regression

This re-weighting technique can make results credible even if the common trend premise is not fully satisfied. Given that LCCs were launched in 2012, and the policy was implemented in 2018 and 2020, we selected data from 2016 and 2018 to observe the short-term health effects of LCCs, and 2016 and 2020 data to investigate their long-term health effects, since the ABSDID method requires two phases of panel data. The results are shown in Table 3.

Except for long-term memory in the 2016 and 2020 data, most of the ATEs were significant. This also showed that the benchmark regression conclusion was reliable even if the common trend assumption was not satisfied. In addition, the outcomes were similar after comparing the benchmark regression results with those of the ABSDID analysis. After comparison, it can also be concluded that the long-term health effects were greater than the short-term effects (Table 3).

#### 4.2.2. Propensity Score Matching

**Figure 2:**
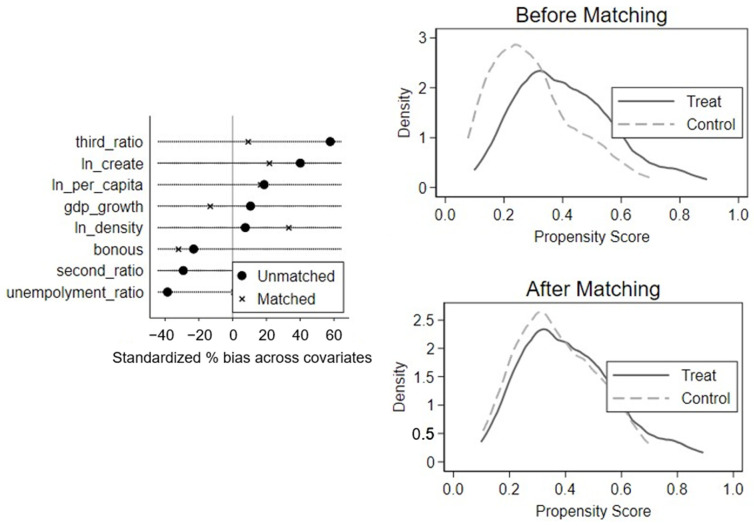
Propensity score matching (PSM).

The upper left corner of Figure 2 shows the balance of covariates and illustrates that the bias of the covariates decreased after using PSM. In addition, the matching optimization of the treatment and control groups largely improved, as can be seen in the kdensity maps, basically satisfying the common trend. We further performed a DID estimation of Equation (1). The results in Table 4 show the estimated results of the PSM-DID.

The estimated results were basically consistent with the benchmark conclusions in terms of significance and coefficients, after considering the systematic differences in the economic and social conditions of the control and treatment groups. To summarize, LCCs were still shown to improve the physical and mental health of elderly people.

## 5. Mechanism Analysis

After empirical testing through a robust analysis, we believed that the construction of LCCs can improve the physical and mental health of elderly residents. How has the construction of these cities enhanced the health of elderly people? Research Hypotheses 1 and 2 predicted that LCCs can improve the health of the elderly population by reducing their probability of being exposed to haze. First, we needed to identify whether the building of LCCs can reduce haze pollution. As with Equation (1), we used a DID identification strategy. The cities were analyzed using the PSM method, which is equal to robustness testing, due to their diversified social and economic conditions.

As shown in Table 5, column (1) only controls for the time and the fixed effect of the city. In general, LCCs reduced haze pollution by 1.8% compared with other cities that had not implemented the policy. After adding the city-level control variables, including the FDI ratio and industrial value-added ratio, the ATE increased slightly. Thereafter, the weather-level control variables were added to the equation, including the air flow coefficient (which can control the haze spillover effect) and other weather variables, and the coefficient significance slightly decreased. Finally, adding the city-level PSM covariates to the regression, the LCCs still reduced haze pollution, although the significance was reduced. It can be concluded that the haze reduction effect of LCCs remained at 2%.

As shown in Table 6, the above results show that after controlling for the city-level variables and the spatial spillover effect of haze, the building of the LCCs still significantly reduced haze pollution. This verified the mechanism whereby LCCs can reduce haze pollution and, thus, enhance the physical and mental health of elderly residents. This conclusion needed to satisfy the common trend test; referring to [41] Beck et al. (2010), Figure 3 shows that the common trend was basically satisfied.

As mentioned above, LCCs can reduce haze pollution. To determine if the policy has long-term reduction effects, we lagged the construction of the LCCs by one and two years. The results shown in columns (4) and (5) were still significant, demonstrating that local government officials continued to make efforts to achieve the declared emission reduction target, which was manifested by a continuous reduction in haze pollution. This result also reveals that the long-term health effect was larger than the short-term effect shown in Table 3. At the same time, we advanced the policy by one year and two years, respectively. The results are listed in columns (1) and (2). We did not find a reduction in haze pollution in the pilot cities compared with the non-pilot cities, which further confirmed the causal effect between LCCs and haze reduction.

After confirming that LCCs can reduce haze pollution, it was necessary to consider the underlying mechanism. The analyses presented in columns (1), (2), and (3) in Table 7 tested whether LCCs can reduce haze pollution by promoting enterprises’ technological innovation. Equation (1) represents the mechanism of GTFP; the average treatment effect was significantly negative. This validated our previous inference that the development of LCCs has led pilot enterprises to accept responsibility for haze reduction.

We also needed to consider whether the demonstrated effect was induced by the improvement of existing GTE or innovations in GTC. Columns (2) and (3) in Table 8 demonstrate that the pilot enterprises had mainly reduced haze pollution in the pilot cities through technological innovations, but not by improving the efficiency of existing technologies. We divided patents into green invention and green utility patents since green patents are regarded as a source of GTC. Columns (3) and (4) reveal that the enterprises’ GTC mainly stemmed from an increase in the number of green utility patent applications.

We partially validated Hypothesis 2, which predicted that enterprises in LCCs would improve the city’s GTC, reducing the probability that the elderly would be exposed to haze pollution, thereby improving their health status. Similarly, column (4) verified Hypothesis 1: after the introduction of LCCs, the government reduced haze pollution by improving the intensity of environmental regulations, thus also improving health indicators among the elderly population.

As posited by Hypothesis 3, a pilot city government may increase the development of green infrastructure and increase public investment in healthcare to improve the health of elderly people. Therefore, following Jann (2014) [42], we used the DID method to draw a series of ATE trend graphs that included green infrastructure construction, such as the urban green space area, the use of renewable energy, and financial investment in public health, which comprised the number of hospitals and vacant hospital beds and health and family planning inputs. Industrial SO_2_ (sulfur dioxide) and industrial sewage emissions were also included.

**Figure 3:**
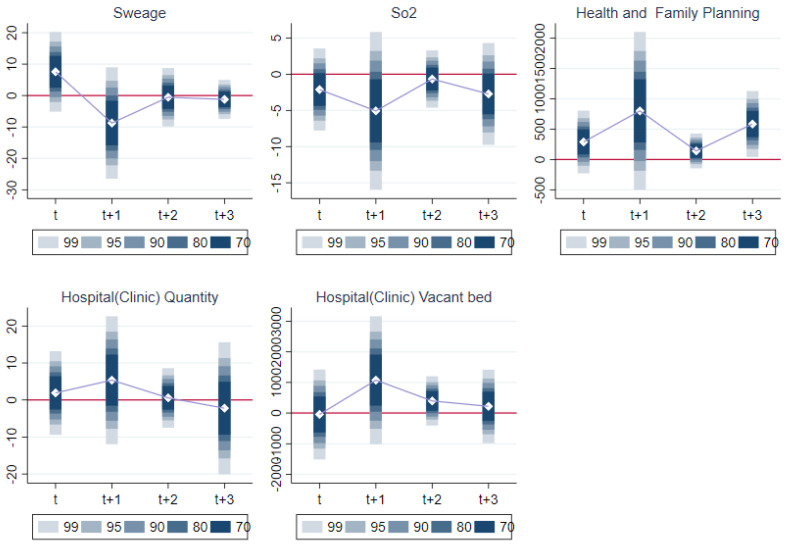
Trend dynamic graph.

Figure 3 shows that industrial SO_2_ and sewage emissions saw a downward trend on average, which also confirms the long-term effect of LCCs on pollution reduction. In addition, compared with non-pilot cities, the number of hospitals and vacant beds and health and family planning inputs in pilot cities significantly increased. Furthermore, as shown in columns (5) and (6) of Table 5, the pilot cities increased their urban green space area and the use of renewable energy more than those cities that had not implemented the policy.

## 6. Conclusions

Using data from the China Health and Retirement Longitudinal Study and the 2012 LCC pilot, this study found that LCC can alleviate haze pollution and improve the health of the elderly. Specifically, the building of LCCs can reduce the blood pressure of elderly people by 1.8% and improve their vital capacity by as much as 14%. The obesity levels of the elderly were also reduced to a certain extent, while their memory increased significantly: short-term instantaneous memory was improved by nearly 12%, and long-term persistent memory was enhanced by 0.8%. The above conclusions remained valid after a series of robustness tests.

The study further investigated how the mechanism of new urbanization can improve the health of elderly people. It found that new urbanization can reduce haze pollution in cities and have a long-term lasting effect, which is still obvious after considering the spatial spillover effect of PM_2.5_. This is achieved by the government increasing the intensity of environmental regulations compliance, which reduces pollution and enhances residents’ health from the perspective of reducing undesirable outputs. Moreover, the construction of LCCs reduces the probability of elderly residents being exposed to haze by increasing the city’s GTFP and the efficiency of GTC, which is brought about by the increasing enthusiasm of firms to apply green utility patents.

Finally, other possible channels for improving residents’ health, such as the government’s green public infrastructure and investment in healthcare, were examined, including the urban green space area, the use of renewable energy, the number of hospitals and vacant hospital beds, and health and family planning inputs. The study concludes that the elderly received health dividends from the improvement of living, transportation, and medical support resulting from the construction of LCCs. In sum, the construction of LCCs has improved the health status of the elderly, who have a high incidence of diseases, from three different perspectives: government financial support and environmental regulations, enterprise technology innovation, and residents’ exercise and travel habits. The current study has been conducted based on data from China, and a comparative study with data from another similar country could further improve the robustness of findings and improve the generalizability of this study.

The world population is aging, with a significant increase in the percentage of people above 60 years old. The aggravation of aging has forced us to face the health problems of the elderly. They represent a segment of the population that is more vulnerable to adverse environmental conditions [43,44]. As an emerging urbanization model, the low-carbon city represents the continuous development of the concept of sustainable development.

This study provides proof that low-carbon cities improve the physical health of the elderly, reflecting the benefits of sustainable development to society, nature, and human health.

## Figures and Tables

**Table 1:** Data sources for primary variables.

Variable	Data Sources
PANEL A: Individual Variables	
Blood Pressure	CHARLS
Weight	
Vital Capacity	
Self-Rated Memory	
Short-Term Memory	
Long-Term Memory	
PANEL B: City Variables	
PM_2.5_	Columbia University
Green Invention Patents	Chinese Intellectual Property Office Patent database
Green Utility Patents	
Green Space	CEIC database
Green Buses	
Hospital Beds	
Hospitals (Clinics)	
Sewage	
SO_2_	
FDI Ratio	
Population Density	
Financial Surplus	
Unemployment Rate	
Secondary Industry Ratio	
Industry Value-Added	
Tertiary Industry Ratio	
Capital Per Capita	
Urban Innovation Index	Fudan University
Temperature	China National Meteorological Administration
Humidity	
Rain	
Sunshine Duration	
Air Flow	European Weather Data Center

**Table 2:** Testing for the difference of grouping means of variables.

Variables	N-Control	Mean (C)	N-Treat	Mean (T)	Difference
Panel A: Micro Dependent Variables					
Blood Pressure	7317	4.620	791	4.650	−0.03 ***
Weight	7368	4.060	795	4.110	−0.05 ***
Vital Capacity	7081	5.450	746	5.280	0.17 ***
Self-Rated Memory	8316	4.130	1029	3.990	0.14 ***
Short-Term Memory	7658	0	884	−0.120	0.12 ***
Long-Term Memory	7658	2.830	884	2.810	0.01 ***
Panel B: Micro Control Variables					
Minimal Exercise	8918	0.320	1090	0.270	0.06 ***
Intensive Exercise	8918	0.150	1090	0.090	0.06 ***
Medium Exercise	8918	0.240	1090	0.190	0.05 ***
Marriage	8918	0.890	1090	0.900	−0.01
Smoking	8918	0.280	1090	0.270	0.01
Drinking	8918	0.330	1090	0.350	−0.01
Family Average Income	8918	2.240	1090	2.810	−0.57 ***
Self-Rated Health	8918	3.490	1090	3.510	−0.02
Age	8918	59.74	1090	59.91	−0.170
Visited Hospital in the Last Month	8918	1.800	1090	1.830	−0.03 **
Hospitalized in the Last Year	8918	1.920	1090	1.910	0
Has Medical Insurance	8918	0.940	1090	0.920	0.02 **
Panel C: City Variables					
PM_2.5_	210	3.530	67	3.460	0.0700
Environmental Regulation	178	0.120	58	0.140	−0.0100
GTFP	159	0.990	58	0.990	−0.0100
GTE	159	1	58	0.980	0.0200
GTC	159	0.980	58	0.970	0.0100
Green Invention Patents					
Green Utility Patents					
Green Space	211	0.0100	69	0.0200	−0.01 **
Green Buses	211	0.540	69	0.720	−0.17 **
Health and Family Planning	211	1323	69	1396	−73.28
Hospital Beds	211	14,384	69	15,740	−1356
Hospitals (Clinics)	211	203.7	69	211.0	−7.310
Sewage	211	78.87	69	83.27	−4.400
SO_2_	211	60.24	69	60.74	−0.500
FDI Ratio	211	0.0200	69	0.0300	−0.01 ***
Population Density	211	1.530	69	1.390	0.140
Financial Surplus	211	−0.540	69	−0.490	−0.0500
Unemployment Rate	211	0.0600	69	0.0400	0.01 ***
Urban Innovation Index	211	0.750	69	1.050	−0.30 **
Secondary Industry Ratio	211	0.510	69	0.500	0.0100
Industry Value-Added	211	1410	69	1471	−60.48
Tertiary Industry Ratio	211	0.350	69	0.380	−0.03 **
Capital Per Capita	211	10.22	69	10.26	−0.0400
Temperature	209	14.18	69	16.11	−1.93 ***
Humidity	209	67	69	71.30	−4.31 ***
Rain	209	1042	69	1306	−263.29 ***
Sunshine Duration	209	1954	69	1884	69.95
Air Flow	209	7.470	69	7.480	−0.0100

Note: *t*-values are reported in parentheses. The robust standard errors are clustered at the city level. ** and *** represent statistical significance levels of 5% and 1%, respectively. Panel A and Panel B are descriptive statistical analyses of the CHARLS data. Panel C is a mix of city-level variables, which includes PM_2.5_, dependent variables, and other macro-control variables.

**Table 3:** Benchmark regression.

	Physical Health			Mental Health		
	Blood Pressure	Vital Capacity	Weight	Self-Rated Memory	Short-Term Memory	Long-Term Memory
DID	−0.018 ***	0.140 ***	−0.017 ***	0.090 ***	−0.116 ***	−0.008 *
	(−3.92)	(5.02)	(−4.59)	(3.17)	(−3.29)	(−1.82)
S-E	0.001	−0.015 *	0.003 **	−0.001	0.006	0.001
	(0.47)	(−1.77)	(2.34)	(−0.04)	(0.39)	(0.69)
I-E	−0.001	0.001	−0.004 **	−0.038 **	−0.021	−0.000
	(−0.48)	(0.14)	(−2.32)	(−2.20)	(−1.02)	(−0.18)
M-E	−0.000	0.008	0.000	0.006	−0.023	−0.001
	(−0.05)	(0.91)	(0.07)	(0.39)	(−1.33)	(−0.62)
Marriage	−0.005	0.076 ***	−0.003	−0.005	−0.049	−0.010 *
	(−0.76)	(3.40)	(−0.77)	(−0.13)	(−1.06)	(−1.81)
Smoking	0.004	0.005	−0.003 **	0.015	−0.007	0.000
	(1.20)	(0.45)	(−2.02)	(0.82)	(−0.35)	(0.10)
Drinking	0.001	0.021 **	0.002	0.017	−0.072 ***	−0.006 **
	(0.52)	(2.11)	(1.15)	(1.03)	(−3.61)	(−2.39)
Income	0.001 *	0.000	0.000	−0.009 ***	−0.003	−0.000
	(1.83)	(0.09)	(0.72)	(−3.49)	(−0.88)	(−0.56)
Health	−0.002	−0.001	−0.000	0.109 ***	0.006	0.001
	(−1.59)	(−0.33)	(−0.03)	(16.93)	(0.93)	(1.51)
Age	0.001 **	0.023 ***	0.001 ***	0.011 ***	−0.007 **	0.005 ***
	(2.25)	(13.90)	(3.37)	(4.00)	(−2.07)	(11.32)
Hospital Visit	0.004 *	−0.006	0.002	−0.028 **	0.039 **	0.003 *
	(1.81)	(−0.71)	(1.33)	(−2.20)	(2.56)	(1.70)
Hospitalized	0.006 **	0.009	−0.001	0.003	−0.025	−0.001
	(2.19)	(0.88)	(−0.34)	(0.19)	(−1.31)	(−0.51)
Medical-I	0.001	0.025 *	−0.003	−0.001	−0.098 ***	−0.005
	(0.22)	(1.73)	(−1.28)	(−0.04)	(−3.62)	(−1.60)
_cons	4.545 ***	3.976 ***	4.015 ***	3.117 ***	0.543 **	2.547 ***
	(146.35)	(35.19)	(209.62)	(16.10)	(2.41)	(88.82)
year fixed	YES	YES	YES	YES	YES	YES
id fixed	YES	YES	YES	YES	YES	YES
N	23266	22,721	23,430	27,790	27,094	27,094
adj. R^2^	0.005	0.029	0.011	0.025	0.003	0.015
F	5.304	23.815	12.394	29.193	4.434	20.482

Note: *t*-values are reported in parentheses. The robust standard errors are clustered at the city level. *, **, and *** represent statistical significance levels of 10%, 5%, and 1%, respectively. DID: LCCs; S-E: minimal exercise; I-E: intensive exercise; M-E: medium exercise; Income: family average income; Health: self-rated health; Hospital Visit: visited hospital in the last month; Hospitalized: hospitalized in the last year; Medical-I: has medical insurance; N: sample size; adj.: adjusted.

**Table 4:** Semi-parametric difference-to-difference (SDID) results.

	Physical Health			Mental Health		
	Blood Pressure	Vital Capacity	Weight	Self-Rated Memory	Short-Term Memory	Long-Term Memory
**Panel A: 11, 13**						
ATE	−0.017 ***	0.112 ***	−0.015 ***	0.096 ***	−0.113 ***	−0.010 *
	(−2.83)	(3.76)	(−3.60)	(2.95)	(−2.70)	(−1.89)
control, year, id fixed effect						
N	6183	5775	6284	8692	8027	8027
**Panel B: 11, 15**						
ATE	−0.022 ***	0.155 ***	−0.020 ***	0.076 **	−0.127 ***	−0.007
	(−4.06)	(4.86)	(−4.64)	(2.36)	(−3.35)	(−1.33)
control, year, id fixed effect						
N	6669	6423	6798	8890	8201	8201

Note: *t*-values are reported in parentheses. The robust standard errors are clustered at the city level. *, **, and *** represent statistical significance levels of 10%, 5%, and 1%, respectively. ATE: average treatment effect; N: sample size.

**Table 5:** Propensity-score-matching (PSM)-DID.

	Physical Health			Mental Health		
	Blood Pressure	Vital Capacity	Weight	Self-Rated Memory	Short-Term Memory	Long-Term Memory
ATE	−0.021 ***	0.136 ***	−0.017 ***	0.084 ***	−0.121 ***	−0.008 *
	(−4.42)	(4.86)	(−4.55)	(2.94)	(−3.40)	(−1.86)
_cons	4.489 ***	3.922 ***	4.016 ***	3.055 ***	0.505 **	2.550 ***
	(139.32)	(33.18)	(205.76)	(15.19)	(2.17)	(85.90)
control	YES	YES	YES	YES	YES	YES
year fixed	YES	YES	YES	YES	YES	YES
id fixed	YES	YES	YES	YES	YES	YES
N	21,747	21,262	21,894	25,875	25,224	25,224
adj. R^2^	0.006	0.031	0.011	0.025	0.003	0.015
F	5.989	23.177	11.833	27.811	4.250	19.357

Note: *t*-values are reported in parentheses. The robust standard errors are clustered at the city level. *, ** and *** represent statistical significance levels of 10%, 5%, and 1%, respectively. ATE: average treatment effect; N: sample size; adj.: adjusted.

**Table 6:** Mechanism analysis.

	(1)	(2)	(3)	(4)	(5)	(6)
	PM_2.5_				Green Buses	Green Space
DID	−0.022 **	−0.026 **	−0.021 **	−0.019 *	0.030 **	0.002 *
	(−2.01)	(−2.28)	(−1.98)	(−1.68)	(2.12)	(1.80)
control	NO	YES	YES	YES	YES	YES
climate fixed	NO	NO	YES	YES	NO	NO
psm control	NO	NO	NO	YES	NO	NO
city fixed	YES	YES	YES	YES	YES	YES
year fixed	YES	YES	YES	YES	YES	YES
N	2493	2380	2033	2012	2378	2374
adj. R^2^	0.962	0.957	0.960	0.960	0.968	0.964
F	4.059	2.455	1.987	3.345	1.723	2.368

Note: *t*-values are reported in parentheses. The robust standard errors are clustered at the city level. * and ** represent statistical significance levels of 10% and 5%, respectively. N: sample size; adj.: adjusted.

**Table 7:** Change treatment year analysis.

	(1)	(2)	(3)	(4)	(5)
	PM_2.5_				
treat(−2)	−0.005				
	(−0.34)				
treat(−1)		−0.010			
		(−0.88)			
treat			−0.026 **		
			(−2.28)		
treat(1)				−0.021 *	
				(−1.90)	
treat(2)					−0.026 **
					(−2.38)
control	YES	YES	YES	YES	YES
city fixed	YES	YES	YES	YES	YES
year fixed	YES	YES	YES	YES	YES
N	2380	2380	2380	2380	2380
adj. R^2^	0.957	0.957	0.957	0.957	0.958
F	0.815	1.007	2.455	1.797	2.478

Note: *t*-values are reported in parentheses. The robust standard errors are clustered at the city level. * and ** represent statistical significance levels of 10% and 5%, respectively. N: sample size; adj.: adjusted.

**Table 8:** Further mechanism analysis.

	(1)	(2)	(3)	(4)	(5)	(6)
PM_2.5_						
DID*ML	−0.071 **					
	(−2.01)					
DID*TC		−0.073 *				
		(−1.79)				
DID*EC			0.005			
			(0.13)			
DID*UTILITY				−0.015 ***		
				(−2.72)		
DID*APPLY					−0.007	
					(−1.50)	
DID*SCORE						−0.066 **
						(−2.56)
control	YES	YES	YES	YES	YES	YES
city fixed	YES	YES	YES	YES	YES	YES
year fixed	YES	YES	YES	YES	YES	YES
N	1862	1862	1862	2380	2380	1994
adj. R^2^	0.949	0.949	0.949	0.958	0.958	0.955
F	3.867	3.516	3.235	5.418	4.065	3.055

Note: *t*-values are reported in parentheses. The robust standard errors are clustered at the city level. *, **, and *** represent statistical significance levels of 10%, 5%, and 1%, respectively. N: sample size; adj.: adjusted.

## Data Availability

The data comes from the CHARLS database and the National Bureau of Statistics of China.
